# Assessment of the In Vivo Release and Biocompatibility of Novel Vesicles Containing Zinc in Rats

**DOI:** 10.3390/molecules26134101

**Published:** 2021-07-05

**Authors:** Liliana Mititelu-Tartau, Maria Bogdan, Daniela Angelica Pricop, Beatrice Rozalina Buca, Ana-Maria Pauna, Lorena Anda Dijmarescu, Ana-Maria Pelin, Liliana Lacramioara Pavel, Gratiela Eliza Popa

**Affiliations:** 1 Department of Pharmacology, Faculty of Medicine, “Grigore T. Popa” University of Medicine and Pharmacy, 700115 Iasi, Romania; liliana.tartau@umfiasi.ro (L.M.-T.); beatrice-rozalina.buca@umfiasi.ro (B.R.B.); ana-maria-raluca-d-pauna@umfiasi.ro (A.-M.P.); 2 Department of Pharmacology, Faculty of Pharmacy, University of Medicine and Pharmacy, 200349 Craiova, Romania; 3 Department of Physics, Faculty of Physics, “Al. I. Cuza” University, 700506 Iasi, Romania; 4 Department of Obstetrics-Gynecology, Faculty of Medicine, University of Medicine and Pharmacy, 200349 Craiova, Romania; lorenadijmarescu@yahoo.com; 5 Department of Pharmaceutical Sciences, Faculty of Medicine and Pharmacy, “Dunarea de Jos” University, 800010 Galati, Romania; anapelin@gmail.com; 6 Department of Morphological and Functional Sciences, Faculty of Medicine and Pharmacy, “Dunarea de Jos” University, 800010 Galati, Romania; doctorpavel2012@yahoo.com; 7 Department of Pharmaceutical Technology, Faculty of Pharmacy, “Grigore T. Popa” University of Medicine and Pharmacy, 700115 Iasi, Romania; eliza.popa@umfiasi.ro

**Keywords:** zinc chloride, lipid vesicles, biocompatibility, rats

## Abstract

This paper is focused on the in vivo release and biocompatibility evaluation in rats of some novel systems entrapping zinc chloride in lipid vesicles. The particles were prepared by zinc chloride immobilization inside lipid vesicles made using phosphatidylcholine, stabilized with 0.5% chitosan solution, and dialyzed for 10 h to achieve a neutral pH. The submicrometric systems were physico-chemically characterized. White Wistar rats, assigned into four groups of six animals each, were treated orally with a single dose, as follows: Group I (control): deionized water 0.3 mL/100 g body weight; Group II (Zn): 2 mg/kg body weight (kbw) zinc chloride; Group III (LV-Zn): 2 mg/kbw zinc chloride in vesicles; Group IV (LVC-Zn): 2 mg/kbw zinc chloride in vesicles stabilized with chitosan. Haematological, biochemical, and immune parameters were assessed after 24 h and 7 days, and then liver fragments were collected for histopathological examination. The use of zinc submicrometric particles—especially those stabilized with chitosan—showed a delayed zinc release in rats. No substantial changes to blood parameters, plasma biochemical tests, serum complement activity, or peripheral neutrophils phagocytic capacity were noted; moreover, the tested substances did not induce liver architectural disturbances. The obtained systems provided a delayed release of zinc, and showed good biocompatibility in rats.

## 1. Introduction

Zinc (Zn) is the second most abundant metal in the human body after iron, being the only metal present in the composition of all types of enzymes and all tissues—mainly in the brain, muscles, bones, kidneys, and liver [1]. Zn is a trace element involved in many body functions, being considered a component part of the tissue antioxidant defence system;it influences cytokine production and stabilizes the cell membrane, preventing inflammatory injuries, thus being considered an antioxidant and an anti-inflammatory agent [2,3].

Zn is part of many processes of cell metabolism; about 10% of proteins bind to Zn, and over 100 proteins transport this divalent cation in the body [4,5]. In mediating the activity of enzymatic systems, Zn is involved in the catalytic activity of over 200 enzymes, such as oxidoreductases, transferases, hydrolases, and ligases [6,7,8]. Two of the enzymes containing Zn (carbonic anhydrase and carboxypeptidase) are vital for the processes of regulating carbon dioxide and protein digestion modulation, respectively [9].

Zn plays a decisive role in immune function, oxidative stress, regulation of apoptotic phenomena, and also in mediating pathological changes related to the aging process [10,11]. The high degree of Zn protection influences cell membrane stability and creates a solid barrier against free radicals. It has been shown that a decrease in Zn levels leads to the induction of oxidative stress. The pathophysiological processes by which this bivalent cation mediates cell protection against the negative action of oxygen free radicals involve the preservation of protein sulfhydryl groups. In addition, as a result of its stabilizing effect on the proteins and lipids, Zn exerts a protective effect on the cell membrane, participating in the degradation processes of the cellular oxidation products [12,13,14].

Zn absorption takes place in the small intestine—especially in the jejunum—and it is mainly excreted through the intestine [15,16]. Vegans/vegetarians, the elderly, and individuals with chronic diseases—such as inflammatory bowel disease or liver cirrhosis—are more prone to Zn deficiency [17,18,19]. Drug therapies such as anticonvulsants, laxatives, antacids, penicillamine, and ethambutol are also contributors to Zn deficiency [20]. According to the WHO, Zn deficiency affects approximately one-third of the global population, and although the severe stage is rare, mild-to-moderate deficiency is more frequent worldwide [21,22]. The highest prevalence is in developing or poor countries, yet the symptoms of Zn deficiency are reversible [22]. Increasing dietary diversity, industrial food fortification, multiple micronutrient powders or supplements, and zinc supplementation are common methods to solve Zn deficiency [23].

Lipid vesicles are spherical entities containing a hydrophilic inner core coated with an outer membrane consisting mainly of phospholipids. These vesicles are biocompatible, biodegradable, non-immunogenic, and non-toxic [24].In their initial state, the vesicles are unstable, with the drug leaking from the inside core during storage [25].To overcome these limitations, the lipid vesicles can be coated with polymers, leading to better stability and longer circulation in the bloodstream. Modifying the outer surface of the vesicles with chitosan by hydrogen bonding with the phospholipids will increase the vesicles’ stability, leading to better storage of the encapsulated drug. Chitosan is a cationic linear polysaccharide, considered to be a valuable biomaterial for drug delivery systems, due to its biocompatibility and low toxicity [26].The aim of our study was to evaluate the in vivo release and biocompatibility of novel submicrometric formulations containing Zn chloride in rats.

## 2. Results

**Figure 1:**
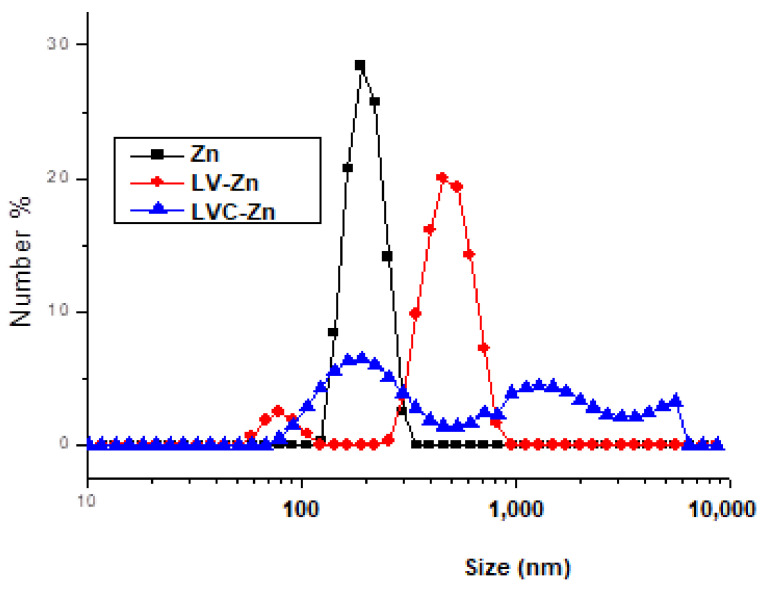
Size distribution frequency of Zn vesicles in solution.

Stability studies were performed to the compare size, homogeneity, and zeta potential of the obtained dispersions. The chart displaying size distribution suggests that the encapsulated drug in lipid vesicles (LV-Zn) has a major population of particles with a mean size of 485 nm. Chitosan addition modified the vesicles’ surface, leading to a stiffening of the vesicles. The measurement of the hydrodynamic size of the chitosan-coated vesicles was carried out after purification and dialysis for 10 h. The size chart evidenced two LVC-Zn populations: the major population, with a mean size of 306 nm, suggests that chitosan leads to a confinement of the vesicles; the minor population, with a mean size of 789 nm, suggests a flocculation effect of the vesicles (Figure 1). The dialysis process induced a decrease in vesicle stability, due to the protonation/deprotonation phenomena.

**Figure 2:**
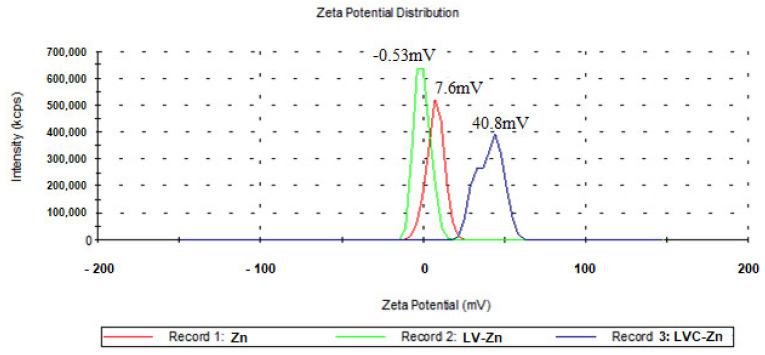
Distribution of the Zn vesicles’zeta potential.

After dialysis, the LVC-Zn vesicles showed moderate-to-good stability (zeta potential of 40.8 mV), while LV-Zn were found in the state of agglomeration (zeta potential of −0.5 mV) (Figure 2). By adding chitosan to the solution of Zn-containing particles, their surface became positively charged, leading to repulsive forces, due to the protonation of chitosan amino groups to the surface of lipid vesicles, thus preventing their agglomeration.

**Figure 3:**
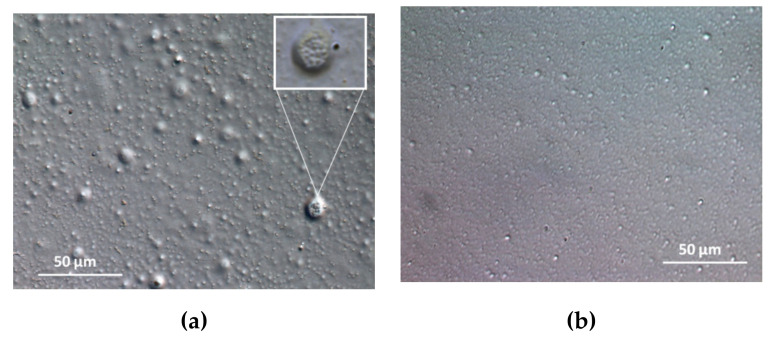
DIC (20×) optical microscopy images representing (**a**) LV-Zn and (**b**) LVC-Zn before dialysis.

The images showed that in the obtained dispersion, the LV-Zn vesicles had different sizes. The largest vesicles consisted of clusters of small vesicles (Figure 3a). Before dialysis, the vesicles entrapping Zn stabilized with chitosan (LVC-Zn) were small and uniformly distributed throughout the liquid;they were of regular shape (close to spherical), and were welldispersed due to stabilization with chitosan, before the dialysis procedure (Figure 3b).

**Figure 4:**
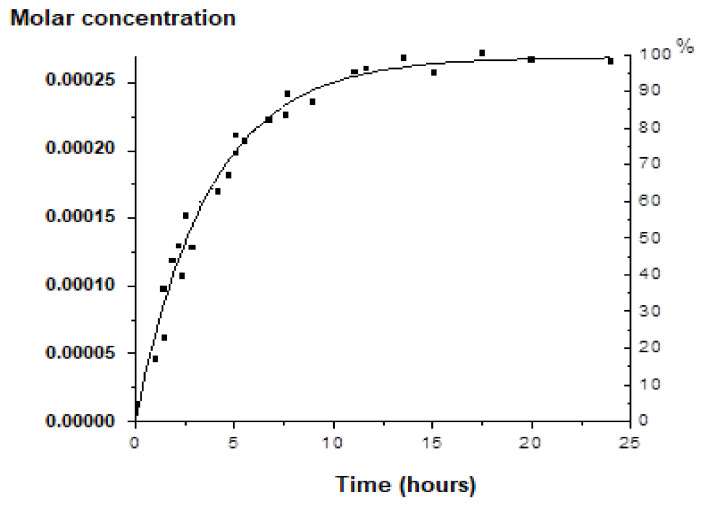
The in vitro release of zinc (molar concentration) from LVC-Zn. Values are expressed as mean ± S.D.

In the in vitro release determination, it can be noticed that the molar concentration of Zn released increased slowly over time, with a complete release from the LVC-Zn at 24 h (Figure 4).

**Figure 5:**
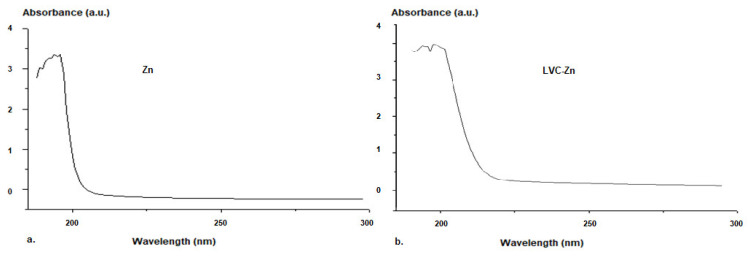
UV–VIS Spectra for Zn (**a**) and LVC-Zn (**b**) in solution.

To determine the loading degree of Zn in the vesicles, ZnCl concentration in the lipid vesicle suspension was measured using UV–VIS spectroscopy, taking the absorption spectrum of zinc chlorideas a reference (Figure 5).

The molar concentration of Zn was determined in vitro using a UV–VIS spectrophotometer (Hewlett Packard 8453, equipped with a HP Chem-Station software) by extracting 2 mL of the release medium at equal 1 h intervals of time. After each determination, the same volume of medium was replaced in the dissolution compartment.

The analysis of the absorption spectrum of Zn chloride showed that the lipid vesicles stabilized with chitosan had a high loading efficiency of Zn. The calibration curve of Zn was linear. The correlation with the etalonation curve indicated a 92% efficiency of Zn entrapment into the lipid vesicles.

**Figure 6:**
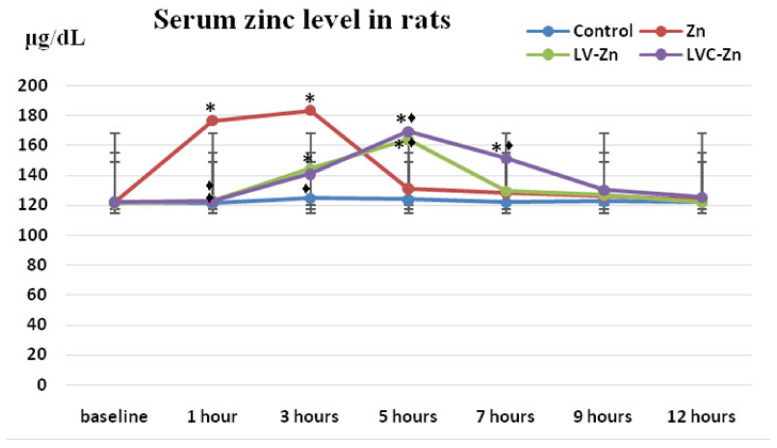
Variation of serum Zn levels after the administration of LV-Zn and LVC-Zn in rats; * *p* < 0.01 vs. baseline; ♦ *p* < 0.01 vs. Zn.

The blood tests of rats at different moments in time showed that in the group that received the non-loaded substance, Zn levels showed a fast and progressive increase—statistically significant compared to baseline—reaching a maximum after 3 h (* *p* < 0.01), and sharply decreasing afterwards, with values similar to the control group after 5 h into the experiment (Figure 6).

LV-Zn administration was correlated with a slow increase in serum Zn levels, which became statistically significant compared to the initial moment in the interval between 3 h (* *p <* 0.01) and 5 h (* *p <* 0.01); after this moment, there was an important decrease in values similar to those measured in the group treated with deionized water (Figure 6).

The use of LVC-Zn was followed by an increase in plasma Zn levels, after a latency period, being statistically significant compared to baseline in the interval of 3–7 h (* *p <* 0.01), with maximum values at 5 h into the experiment. After this interval, the Zn levels abruptly decreased, reaching values close to those of the deionized water group. In the group treated with LVC-Zn, the serum Zn concentration was higher than in the group that received LV-Zn at all moments of determination in the experiment (Figure 6).

The in vivo studies revealed no considerable changes in the percentage values of the leucocyte formula elements—polymorphonuclear neutrophils (PMN), lymphocytes (Ly), eosinophils (E), monocytes (M), and basophils (B)—between the Zn, LV-Zn, LVC-Zn, and control groups—neither after 24 h, nor after 7 days into the experiment (Table 1).

Laboratory investigations did not evidence substantial variations in the aspartate transaminase (AST), alanine aminotransferase (ALT), and lactate dehydrogenase (LDH) activity between the Zn, LV-Zn, LVC-Zn, and control groups—neither 24 hnor 7 days after the administration of the tested solution (Table 2).

The administration of Zn, LV-Zn, and LVC-Zn was not associated with perceptible changes in the blood urea and creatinine values compared to the control group at the timepoints of the determinations (Table 3).

The use of Zn, LV-Zn, and LVC-Zn did not determine important dissimilarities in the serum complement activity or the phagocytic capacity of peripheral neutrophils, compared to the group treated with deionized water—neither after 24 h, nor after 7 days, of the experiment (Table 4).

**Figure 7:**
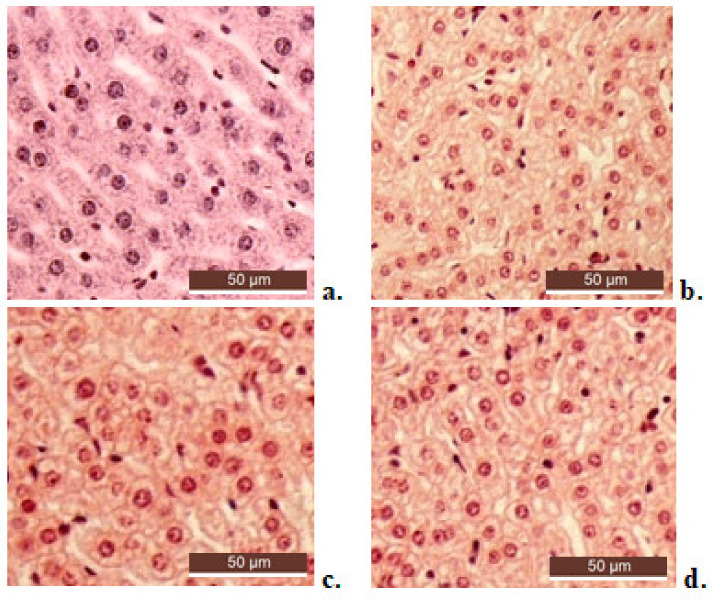
Histological liver architecture in animals treated with deionized water (**a**), Zn (**b**), LV-Zn (**c**), and LVC-Zn (**d**); H&E stain ×10.

No important pathohistological modifications in the liver structure of animals treated with Zn chloride were noted (Figure 7b) compared with the deionized water group (Figure 7a). The histological analysis performed with a light microscope on the liver tissue samples obtained from the LV-Zn (Figure 7c) and LVC-Zn (Figure 7d) groups did not reveal substantial changes in the normal tissue architecture compared to control animals.

## 3. Discussion

Nanotechnology is an extremely attractive domain of research, promising to provide a vast number of innovative applications in various fields of activity, including medicine and biology [27,28]. Vesicles, as submicron-sized carrier systems, are valuable therapeutic tools, generally designed to entrap different compounds, to easily cross biological barriers and penetrate hard-to-reach areas of the body, so as to improve the transport and delivery of the active substances to target tissues [29,30,31].

Over time, much effort has been made to develop nanomaterials containing bivalent cations for various uses in medicine, biology, chemistry, and cosmetics [32,33,34]. Although their particularities have been obviously revealed by size, zeta potential, and chemical composition, their degree of entrapping the drugs, biocompatibility, and toxicity in laboratory animals are still not well established [35,36].

Recently, the development and medical use of nanoparticles containing Zn salts has aroused increasing interest [37,38,39]. Several groups of researchers have focused their attention on obtaining and studying nanoparticles containing Zn oxide, on the premise that this agent has notable antibacterial activities [40,41]. Barreto et al. obtained nanoparticles containing Zn oxide coated with chitosan following a classic ultrasonic method. The physico-chemical characterization revealed the small size of the nanostructures, a good homogeneity of the colloidal dispersion, and antibacterial properties against *Escherichia coli* and *Staphylococcus aureus* [42]. Yusof et al. synthesised Zn oxide nanoparticles stabilized by chitosan using a microwave heating procedure; they reported that nanoparticle size improved with the intensification of power and heating time, and with the stabilization of the solution using chitosan. Moreover, chitosan/Zn oxide nanoparticles showed substantial antibacterial activity against *Staphylococcus aureus* and *Escherichia coli* species [43].

In our study, novel lipid vesicles loaded with Zn chloride were prepared and stabilized. By coating them with the biocompatible and biodegradable polymer chitosan, the vesicles’ size decreased, and their stability increased as a result of the positive charge load.

The biocompatible polymer chitosan has valuable intrinsic properties, such as a lack of in vivo toxicity, great stability, and biodegradability by bodily enzymes [44,45,46];it stabilizes the drug-loaded vesicles in dispersion and plays an important role in absorption efficiency [47].

The soft vesicle surface is electrically charged, as the result of protonation/deprotonation processes that are dependent on the pH of the solution. Comparing the average values of the zeta potential with the criteria of colloid solutions’ stability behaviour, we distinguished significant differences between the analysed samples [48].

The use of Zn particles—especially those stabilized with chitosan—was accompanied by a delayed release of Zn in rats. The delaying of the onset of action and the prolongation of both in vitro and in vivo Zn delivery can be attributed to the release characteristics of the drug entrapped in the lipid vesicles stabilized with chitosan.

Other researchers have studied the effects of nanoparticles based on Zn oxide and chitosan on liver and kidney function, as well as on oxidative stress in laboratory animals; however, the reported results were contradictory. Siba et al. reported the obtaining of Zn oxide nanoparticles—produced by precipitation and the polyol method—with antioxidant properties and important antibacterial effects against some types of Gram-positive and Gram-negative pathogenic strains, such as *Streptococcus mutans, S. pyogenes*, *Vibrio cholerae, Shigella flexneri,* and *Salmonella typhi* [49]. In contrast to these observations, Hajinezhad demonstrated that the administration of Zn oxide/chitosan nanovesicles produced pro-oxidant effects and increased liver enzyme activity, but did not influence renal parameters [50], while other researchers have shown hepatoprotective properties of Zn oxide nanoparticles [51] or alterations in blood kidney functional markers following their administration [52].

In a recent study, the authors found that Zn-gluconate-loaded chitosan nanoparticles produced via anionic gelation method decreased the inflammatory process and the associated oxidative stress, and improved local histological changes, in rats with experimental-collagen-induced rheumatoid arthritis [53].

Our study succeeded in setting an optimal ratio between lipid/chitosan concentrations used to encapsulate ZnCl_2_. The dispersions of drug-loaded vesicles were obtained following classic procedures, through physical methods, without chemical reagents or thermal treatments. Thus, the biocompatibility of the systems was not influenced. Consequently, the vesicles were more stable after dialysis, and could be stored for 3 months at room temperature.

## 4. Materials and Methods

### 4.1. Substances

Zn chloride (≥99%), l-α-phosphatidylcholine ≥ 99% (TLC), chitosan, acetic acid, and deionized water were purchased from Sigma-Aldrich Chemical Co. (Steinheim, Germany). The polysaccharide chitosan with an average molecular weight of 114,374 g/Mol, a deacetylation degree of 79.7%, and a polydispersity index of 3.76, was dissolved in 1% (*v*/*v)* acetic acid solution to obtain 0.5% (*w*/*w*) homogeneous solution. All solutions were made with deionized water.

### 4.2. The Obtaining of Zn Submicrometric Vesicles

The lipid vesicles were prepared by dissolving 15 mg phosphatidylcholine in chloroform; afterwards, the solvent was removed by evaporation, and a dry lipid film was obtained. The film was then hydrated using a solution of Zn chloride (0.014 g Zn chloride in 50 mL of deionized water) and then subjected to ultrasonication for 20 min (amplitude modulus 10% at a 20 kHz ± 500 Hz frequency, Sonoplus Bandeline configuration). Finally, the vesicles were mixed with a solution of 0.5% chitosan, with a ratio of 3:2 lipid vesicles: chitosan (*v/v*) solution.

After preparation, the lipid–Zn chloride–chitosan vesicles were dialyzed for 4 hto remove the acidity and provide a neutral pH [54,55]. For this procedure, tubular dialysis fibre membranes were used (from Sigma-Aldrich Chemical Co., Steinheim, Germany), with a pore size of 12,000 Da MWCO type Sigma D6191-25EA. The pH values of solutions were assessed using a Sartorius Professional PP-50 pH Meter (Sartorius Lab Instruments GmbH & Co. KG, Göttingen, Germany).

### 4.3. The Characterization of Zn Submicrometric Vesicles

The Zn chloride vesicles were physico-chemically and structurally analysed using a Malvern Zetasizer Nano ZS ZEN-3500 apparatus (Malvern Panalytical Ltd, Worchestershire, UK). The lipid vesicles were visualized with an Nikon Eclipse Ti-E inverted microscope (Nikon Solutions Co.,Tokyo, Japan) provided with NIS Elements Basic Research imaging software and a Coolpix 950 digital camera, and images were processed using adigital image correlation (DIC) system. The transmission spectra were recorded using a Hewlett Packard 8453 UV–VIS spectrophotometer (Agilent Technologies Sales & Services GmbH & Co. KG, Waldbronn, Germany), in order to reveal the encapsulation of Zn in vesicles and to assess their release profiles.

### 4.4. The In Vitro Release of Zn from the Submicrometric Vesicles

To determine the loading degree of Zn in the vesicles, ZnCl concentration in the lipid vesicle suspension was measured using UV–VIS spectroscopy, taking the absorption spectrum of zinc chlorideas a reference. The molar concentration of zinc was determined in vitro using a UV–VIS spectrophotometer (Hewlett Packard 8453, equipped with a HP Chem-Station software, Waldbronn, Germany), by extracting 2 mL of the release medium at equal 1 h intervals of time. After each determination, the same volume of medium was replaced in the dissolution compartment. The variation in zinc concentration in the release medium was examined by determining the absorbance (197.6 nm wavelength), indicating the specific maximum absorption peak of its spectrum. The calibration curve of absorbance vs. zinc concentration was also established. The release medium volume was considered to be constant overtime (a negligible evaporation of the solvent).The release rate (*R*) was calculated as the number of moles of zinc released per unitof time and per unit of volume of release medium:

*R* (*t*) = 1/V × dν(*t*) (1)

where V is the volume of the medium, and dν is the number of moles of zinc released during d*t*.

The molar concentration at a certain moment in time (*t*) was calculated using the following formula:

C (*t*) = ν(*t*)/V (2)

where C is the number of moles of zinc concentration in the release medium, and ν is the number of moles of zinc at time *t* in the volume V.

### 4.5. The In Vivo Release of Zn fromSubmicrometric Vesicles

The study was carried out on white, non-genetically-modified, specific-pathogen-free, healthy Wistar rats (10 weeks old, weighing 200–250 g), equally distributed by sex, purchased from the “Cantacuzino” National Medical-Military Institute for Research and Development, Baneasa Station, Bucharest, Romania.

We randomly set 4 groups of 6 rats each, without using inclusion–exclusion criteria for the animals. The substances were administered orally (using an esogastric tube), in a single dose, according to the following protocol:

▪ Group I (control): deionized water 0.3 mL/100 g body weight;
▪ Group II (Zn): 2 mg/kg body weight (kbw) Zn chloride;
▪ Group III (LV-Zn): 2 mg/kbw Zn chloride entrapped in soft vesicles;
▪ Group IV (LVC-Zn): 2 mg/kbw Zn chloride entrapped in soft vesicles stabilized with chitosan.

Blood levels of Zn in rats were measured at baseline (before the administration of the tested substances) after 1 h, 3 h, 5 h, 7 h, 9 h, and 12 h of the experiment.

Zn plasma concentration was determined by atomic absorption spectrophotometry using a PerkinElmer A Analyst 600 Atomic Absorption Spectrometer with graphite furnace and autosampler model AS-800 (PerkinElmer, Woonsocket, RI, United States). A Zn hollow-cathode lamp(HCL)(PerkinElmer, Woonsocket, RI, United States) was used to assess the serum Zn levels. The working parameters used were: λ=213.9 nm; current = 15 mA; energy = 40. For the standard curve,1 μg/L of the working Zn stock solution (Merck Company, Kenilworth, NJ, United States) was used. Heparinized plasma samples (1:200 dilution, 0.02 mL serum + 3.98 mL double-distilled water) were used. A volume of 5 μL of matrix modifier (0.17 mL Mg (NO3)_2_ + 9.83 mL double-distilled water) was used for each sample.

### 4.6. Biocompatibility Evaluation of Submicrometric Vesicles Entrapping Zn

We estimated the biocompatibility properties of Zn chloride vesicles by assessing the leukocyte formula, the activity of liver enzymes, the levels of serum urea and creatinine, the phagocytic capacity of peripheral neutrophils (nitro-blue tetrazolium-NBT test), and serum complement activity, 24 h and 7 days after the administration of the tested substances [56,57].

At 24 h and 7 days after the administration of the studied substances, the rats were anesthetized with ethyl ether, and positioned for contention in a plexiglass device. The animals’ tails were introduced to water heated to 40 °C, so as to dilate the blood vessels in order to collect samples for laboratory examination [58,59]. The tails were placed in a straight position, and one of the lateral veins of each tail was identified; local anaesthesia was performed with a 1% benzocaine solution, at approximately 3 cm distance from the tip of the tail. Subsequently, the area was disinfected with 70% alcohol, and the vein was punctured with a needle tilted to a 20 degree angle, in order to collect a blood sample of 0.2–0.3 mL [60,61]. A gentle pressure was applied after removing the needle, in order to quickly interrupt the blood flow.

Liver tissues were collected for histopathological analysis. The tissue fragments were fixed in a 10% formaldehyde solution, embedded in paraffin wax, and then cut intothin sections (5 μm). The slides were stained with hematoxylin and eosin (H&E), and further examined using a Nikon Eclipse Ti-E inverted microscope (Nikon Solutions Co.,Tokyo, Japan) equipped with a digital image acquisition system.

The research protocol was approved and implemented according to the recommendations of the Committee for Research and Ethical Issues from “Grigore T. Popa” University of Medicine and Pharmacy in Iasi, Romania, in compliance with the European Directive 2010/63/EU regarding the handling of lab animals (Protocol no. 19157/19.10.2009) [62].

### 4.7. Statistical Analysis

The data were expressed as mean +/− standard deviation (S.D.) of mean, and processed using SPSS 17.0 software for Windows using the one-way ANOVA method. *P* (probability)-values below 0.05 were considered to be statistically significant versus control.

## 5. Conclusions

We prepared novel carrier systems entrapping Zn chloride inside lipid vesicles stabilized with chitosan. The obtained vesicles proved to entrap Zn effectively and showed moderate-to-good stability in solution, with the vesicles remaining stable for more than 3 months at room temperature due to an optimal lipid: chitosan concentration ratio. Entrapping of Zn into vesicles delayed both the in vitro and in vivo release of Zn.

Laboratory tests revealed that LVC-Zn administration resulted in comparable haematological, biochemical, and immune responses to Zn and LV-Zn in rats, thus indicating good in vivo biocompatibility. The liver architecture of rats was not significantly altered after treatment with Zn, LV-Zn, or LVC-Zn. All of these results suggest that the designed particles encapsulated in lipid vesicles coated with chitosan may be appropriate for in vivo use as drug delivery systems, with no need for storage in special conditions or at low temperatures.

## Figures and Tables

**Table 1:** The influence of LV-Zn and LVC-Zn on the percentages of the leucocyte formula elements. Values were expressed as mean ± S.D. of mean for 6 animals in a group.

Groups		Leucocyte Formula (% Values)				
		PMN	Ly	E	M	B
**Control**	24 h	29.3 ± 5.45	66.5 ± 9.47	0.6 ± 0.1	3.4 ± 0.05	0.2 ± 0.1
	7 days	29.8 ± 4.37	65.8 ± 10.29	0.7 ± 0.1	3.5 ± 0.1	0.2 ± 0.05
**Zn**	24 h	29.4 ± 4.83	66.0 ± 10.45	0.8 ± 0.05	3.6 ± 0.05	0.2 ± 0.1
	7 days	29.6 ± 5.29	66.1 ± 9.63	0.6 ± 0.05	3.5 ± 0.05	0.2 ± 0.05
**LV-Zn**	24 h	29.5 ± 5.17	66.2 ± 11.17	0.7 ± 0.1	3.4 ± 0.1	0.2 ± 0.05
	7 days	29.7 ± 5.33	65.7 ± 10.33	0.8 ± 0.05	3.6 ± 0.1	0.2 ± 0.05
**LVC-Zn**	24 h	29.4 ± 4.65	66.0 ± 9.81	0.8 ± 0.1	3.6 ± 0.1	0.2 ± 0.1
	7 days	29.8 ± 5.52	66.3 ± 10.55	0.6 ± 0.1	3.5 ± 0.05	0.2 ± 0.1

PMN—polymorphonuclear neutrophils, Ly—lymphocytes, E—eosinophils, M—monocytes, B—basophils.

**Table 2:** The influence of LV-Zn and LVC-Zn on the AST, ALT, and LDH activity. Values were expressed as mean ± S.D. of mean for 6 animals in a group.

Groups		AST (U/mL)	ALT (U/mL)	LDH (U/mL)
**Control**	24 h	43.5 ± 7.55	96.5 ± 14.27	342.38 ± 61.55
	7 days	44.8 ± 7.39	97.2 ± 15.19	344.29 ± 57.67
**Zn**	24 h	42.9 ± 8.83	97.4 ± 14.33	343.67 ± 59.83
	7 days	43.3 ± 7.17	98.1 ± 13.64	344.45 ± 60.64
**LV-Zn**	24 h	43.6 ± 8.72	96.7 ± 15.39	343.52 ± 62.33
	7 days	45.1 ± 6.33	97.3 ± 15.72	347.43 ± 61.45
**LVC-Zn**	24 h	42.9 ± 6.64	97.9 ± 14.45	344.21 ± 59.72
	7 days	44.2 ± 7.43	98.5 ± 13.27	346.17 ± 58.83

AST—aspartate transaminase, ALT—alanine aminotransferase, LDH—lactate dehydrogenase.

**Table 3:** The influence of LV-Zn and LVC-Zn on the serum urea and creatinine concentrations. Values were expressed as mean ± S.D. of mean for 6 rats in a group.

Groups		Urea (mg/dL)	Creatinine (mg/dL)
**Control**	24 h	36.5 ± 5.29	0.05
	7 days	38.7 ± 4.65	0.13
**Zn**	24 h	37.1 ± 5.33	0.11
	7 days	39.3 ± 6.17	0.15
**LV-Zn**	24 h	37.4 ± 5.83	0.07
	7 days	38.5 ± 5.37	0.15
**LVC-Zn**	24 h	36.4 ± 4.19	0.07
	7 days	37.2 ± 5.43	0.09

**Table 4:** The influence of LV-Zn and LVC-Zn on serum complement levels and the NBT test.Values were expressed as mean ± S.D. of mean for 6 rats in a group.

Groups		Complement	NBT Test
**Control**	24 h	16.33 ± 3.29	52.19 ± 8.64
	7 days	17.45 ± 3.33	54.25 ± 7.17
**Zn**	24 h	17.67 ± 4.17	52.64 ± 8.55
	7 days	17.55 ± 3.64	55.33 ± 6.83
**LV-Zn**	24 h	17.29 ± 3.83	53.42 ± 7.29
	7 days	17.74 ± 4.45	54.83 ± 6.67
**LVC-Zn**	24 h	16.17 ± 3.67	52.67 ± 7.33
	7 days	16.83 ± 3.37	53.34 ± 7.64

## Data Availability

Not applicable.

## Sample Availability

Samples of the compounds are not available from the authors.
